# Nurses’ perspectives of the nursing documentation audit process

**DOI:** 10.4102/hsag.v24i0.1121

**Published:** 2019-10-17

**Authors:** Mokholelana M. Ramukumba, Souher El Amouri

**Affiliations:** 1 Department of Health Studies, University of South Africa, Pretoria, South Africa; 2 Al Rahba Hospital, Abu Dhabi, United Arab Emirates

**Keywords:** assessment, audit process, evaluation, nursing documentation, quality initiative

## Abstract

**Background:**

Nursing has an obligation to the public to develop measures for the quality of care to enhance patient safety and efficiency of the system. The first hospital to introduce the clinical audit of nursing documentation was in Abu Dhabi. The rationale was the recognition of the link between clinical audits and the quality of patient care and safety. This article recognises the importance of documentation audits in nursing practice and the role of nurses related to conducting audits in a selected hospital in Abu Dhabi. Many studies have shown the potential benefits of documentation audits to evaluate or assess the quality of recorded nursing assessments and care.

**Aim:**

The aim of this study was to explore nurses’ perspectives of the documentation audit process.

**Method:**

The study adopted an exploratory, descriptive qualitative approach using the evaluation method. Data were collected using three focus group interviews consisting of 4 informatics and 13 documentation link nurses involved in the implementation of the clinical audit on nursing documentation in the selected hospital. Thematic analysis was used to analyse the data.

**Results:**

Three major themes evolved from the research findings: implementation of documentation audit, evaluation of audit and measures to improve documentation audit. Strengths and weaknesses of the documentation audit were articulated by the nurses. Generally, nurses were satisfied with the audit process and made recommendations on improvements.

**Conclusion:**

Processes adopted by the team were reasonable and useful, and the preparation and planning for the clinical audit were regarded as areas of strength. Areas of weaknesses in the implementation processes identified included dissemination of findings and executing improvements. This could be improved with necessary support from the hospital management, especially with regard to release time to implement required changes. The complexity of auditing electronic versus paper-based nursing documentation is acknowledged.

## Introduction

A clinical audit is a quality management tool defined as a systematic process to review patient care against defined and agreed criteria in order to identify practice gaps (Sinni, Cross & Wallace 2011). The medical record is the principal source of information transfer between different healthcare professionals; it facilitates communication and establishes record of care provided to avoid adverse side effects (Dehghan et al. 2013; Walker 2012). Therefore, medical records need to be comprehensive and accurate to support the quality of care provided as well as to support the ethical and legal aspects of care provided (Björvell, Thorell-Ekstrand & Wredling 2000). In this study, the focus is on nursing documentation audit because documentation audits provide opportunities to demonstrate the quality of care (Mykkänen, Saranto & Miettinen 2012).

Nursing has an obligation to the public to develop measures for the quality of care to enhance patient safety and efficiency of the system (Müller-Staub et al. 2009). Therefore, the documentation audit process developed needs to be rigorous, comprehensive, practical and usable (Blake-Mowatt, Lindo & Bennett 2013; Setz & D’Innocenzo 2009). However, literature shows high variability in audit processes (Johnson, Jefferies & Langdon 2010; Wang et al. 2011). Although completing audits is a common activity by clinicians, there are few examples published about how to construct tools for evaluating complex clinical assessment (Ritchie et al. 2014).

### Background of the study

Nursing leaders in this hospital initiated strategies to improve the standard of documentation by assigning a task team to develop a documentation audit process. The intention was to standardise audits to capture all aspects of documentation, with clear guidelines and instructions. The dominant form of documentation in this hospital is electronic. The electronic health record (EHR), *Malaffi* [my file] as it is called in Arabic, maintains clinical information electronically. Malaffi has many applications and forms. For instance, Downtime PowerChart Local Access (PCLA) is an application that provides a snapshot of inpatient chart data during downtime. AdHoc chart is a folder within Malaffi system that contains a collection of forms to document assessments and interventions; PowerForm is one of the forms within the AdHoc folder. All nurses need to know what, when and how to document. The argument advanced for the EHR is that it will improve the quality of documentation and efficiency (Wang et al. 2011). Yet, nursing data are stored in different fields, as shown in the folders within Malaffi, and nurses have to use EHR navigation functionality to document on different folders and fields (Al Baloushi & Ramukumba 2015).

Introduction of the EHR has led to transformation of documentation (Wang 2011). Accurate nursing records is one of the requirements to meet the standards of agencies and accredited organisations. The selected hospital in Abu Dhabi was affiliated with Johns Hopkins Medicine and is accredited by the Joint Commission International Accreditation (JCIA).

Using the auditing process can identify any gap present in documentation as well as improve patient care. However, attention should be given to the frequency of audits and time needed to do it, as it might interfere with staff workload and delay the identifying strategies to address deficiencies (Anderson, Mokracek & Lindy 2009). The hospital documentation guidelines were developed in 2014 to define the minimum expected documentation requirements for nursing staff. They are also provided instructions on what must be documented and the expected standard minimum documentation intervals. The guidelines include several assessments such as admission assessment, pain assessment and reassessment, vital sign monitoring, intake and output monitoring, progress notes, functional screening, nutritional screening, psychosocial assessment, fall risk, skin integrity, discharge planning, daily safety checks and nursing care. The quality aspects addressed by the guidelines are timeliness, accuracy and completeness. These guidelines are augmented by SEHA quality protocols. SEHA is an independent health services company that manages healthcare facilities in the Emirate of Abu Dhabi. Currently, there is no empirical evidence that electronic nursing documentation improves time management and information handling or increases quality of documentation and quality of care (Meibner & Schnepp 2014). Consequently, it is imperative to elicit nurses’ views regarding the audit processes used in contexts where documentation is digitalised.

### Problem statement

The selected hospital mainly uses electronic nursing documentation. The documentation team led by nurses developed the clinical audit process to measure the quality of assessment documentation in accordance with the requirements of the accreditation agency. This hospital was the first in the region to initiate a standardised audit system; there were no benchmarks or models to follow. The audit process was implemented and the results from the audits showed some improvement in the quality of the assessment documentation. However, the accreditation agency identified gaps in the documentation audit processes. In addition, nurses raised concerns regarding the structure of the audits and some believed that the processes could be improved. It was imperative to explore the perspectives of the nurses involved in the documentation audit regarding the audit implementation processes in this hospital in order to identify the gaps. Documentation audits are necessary to ensure patient safety. Therefore, a critical appraisal of their quality monitoring systems was required (Anderson et al. 2009).

### Aim of the study

The aim of this study was to explore the nurses’ perspectives on the audit process used to assess the quality of electronic nursing documentation in a selected hospital in Abu Dhabi. The study intended to find answers for the following research questions:

- What are nurses’ perspectives of the documentation audit in this hospital?
- What measures could be employed to improve the audit process in the selected hospital?

## Materials and methods

### Study design

The study utilised an exploratory, descriptive qualitative approach using the evaluation method. The evaluative case study is defined by Patton (2003) as an inquiry into an event by either an individual or an organisation. It is produced through systematic data collection, analysis and reporting. The evaluative case study was the preferred study design for this study, and it allowed exploration of nurses’ perceptions regarding the meaning of documentation audit and processes involved in an audit. The case in this study referred to an audit process adopted at a selected hospital in Abu Dhabi, and the unit of analysis was nurses’ views about their current audit practices (Bamberger et al. 2004).

### Sampling and sample

The study used the non-probability, purposive sampling method to select participants. As this was an evaluative case study, the approach integrated elements of typical case, homogenous and criteria sampling. The inclusion criterion was link documentation nurses, which is a team of nurses involved in quality management initiatives in the hospital. The sample of the study consisted of 17 nurses, 4 of them were informatics nurses and 13 were link documentation nurses. The purpose was to select participants who were knowledgeable about the audit processes and documentation guidelines and would be in a position to provide rich data on the audit processes in this hospital. In this article, link documentation nurses are referred to as ‘nurses’.

### Data collection

The audit process consists of five stages which shaped the conceptual framework of the study. The study used the following stages as a framework for data collection: stage 1: preparing for the audit; stage 2: selecting audit criteria; stage 3: measuring of performance; stage 4: making improvements; stage 5: sustaining improvement. Data were collected in 2015 using a semi-structured interview, inferring that it will provide an opportunity to allow the participants to talk about their perspectives of implementing the documentation audit. Three focus groups interviews comprising five to six participants in each were conducted. Focus groups are suitable where group interaction amongst participants has the potential to yield greater insights, which can be developed through discussion. The advantage of focus groups is that the views can be compared between participants. A scribe took notes during interviews, and no digital recording was performed. The main question that directed the interviews was, ‘what are your views regarding the documentation audit process in this hospital?’ This was followed by several probing questions focused on the study objectives. Data collection continued until no new information was emerged, signifying the data saturation.

### Data analysis

After conducting the interviews, the researchers studied the recordings of interviews and transcribed them verbatim. The thematic analysis followed steps described by Creswell (2014). These included transcription, immersion in data, coding, developing categories and comparison across categories. Transcriptions were read several times by the authors to obtain a general understanding of the data. Sections of the data which seemed to be distinct opinions of participants were highlighted to develop broad topics, which were abbreviated into the predetermined code. Coded sections were read again to mark sections that fitted into the topic and grouped similar data from the quotes and classified them to develop themes, subthemes and categories (Creswell 2014).

## Trustworthiness and integrity of the study

Trustworthiness reflects the degree of confidence of qualitative researchers in their data. This was assessed using the criteria of credibility, dependability, confirmability and transferability. Credibility was enhanced through prolonged interactions with participants. Participants were provided the opportunity to validate the preliminary findings, including the themes. Two experts in documentation and quality management department reviewed the interview guide for clarity and accuracy. The collected data reflected the voice of the participants. Continuous checks were built into data collection processes by using participants’ verbatim accounts. Thick descriptions of the research methods and data were produced to ensure dependability. To maintain confirmability, the researcher ensured that the interpretation of results was the reflection of the participants’ voice and conditions of inquiry, not the researcher’s perspectives. The in-depth accounts of the documentation audit processes were generated on the premise that, in similar contexts and conditions, the results could be transferable.

### Ethical considerations

Permission, approval and consent for the project were obtained from the hospital’s ethics committee and the documentation task force. Prior to data collection, the researchers met with the documentation task force to explain the purpose of the study and the methodology that will be utilised. It was clarified that the study was not an attempt to pass judgement regarding the quality of audit processes; the appreciative nature of the evaluation was communicated to the participants. They were assured of anonymity and protection from any form of harm. They were also informed of their rights regarding autonomy, privacy, confidentiality and the right to withdraw from the study at any time.

## Results

The data from the interviews produced 3 themes and 11 sub-themes (Table 1).

**TABLE 1:** Nurses’ perspectives and experience in documentation audit.

Main themes	Sub-themes
Implementation of audit process	Quality management initiative Preparing for audit Dealing with uncertainty Selecting the criteria Measuring performance Making improvements Sustaining improvements
Evaluation of audit process	Perceived successes Perceived challenges
Measures to improve documentation audit	Training programmes Provision of adequate resources

### Theme 1: Implementation of audit process

Participants shared their views and experiences of the documentation audit process in the hospital. They believed that documentation audit is a significant, ongoing quality improvement initiative to ensure patient safety. Data from interviews indicated that the participants were conversant with the hospital quality management protocols as well as other regulatory protocols within the Emirate of Abu Dhabi and the country. They were able to relate audit to the hospital quality assurance initiatives: ‘our approach to audit was informed by the hospital strategic decision to be the leading health care facility in the region’ (N12, female, 35 years). Another participant said, ‘the nurse managers’ goal is to embrace the evidence-based model of care and also the accreditation agencies’ expectations’ (N13, female, 25 years).

In this hospital, the preparation for the audits involved the establishment of an audit documentation task force that comprised link and health informatics nurses and other representatives from different units. Its mandate included developing a comprehensive, singular process of clinical audit for the hospital to identify gaps in the nursing documentation in order to improve quality of care and patient outcomes. In order to conform to the requirements of the accreditation agency, the team aimed to assess core nursing documentation, which included admission assessment, ongoing assessment, pain assessment and reassessment, vital sign monitoring, intake and output monitoring, progress notes, functional screening, nutritional screening, psychosocial assessment, fall risk, skin integrity, discharge planning, daily safety checks and nursing care plan. The following were some of their sentiments: ‘we used admission and progres reports as data sources’ (N1, female, 34 years).

According to the participants, the kind of audit they embarked on was a new initiative in this hospital and region, and they had no other local models to learn from. Although they understood the need for audits, they felt unprepared and unsure of the specifics of the audit process. They had to deal with uncertainty and feelings of apprehension whether they would develop a valid and reliable audit process. As one participant mentioned: ‘I was confused and did not know what to do or where to start first, I knew we needed to look for evidence and start preparing for the audit’ (N17, female, 30 years). Another participant added: ‘we had to take the hard route and quickly familiarise ourselves with the procedures of clinical audit to improve patient care’ (N8, female, 42 years). One also remarked: ‘we had members who attended SEHA meetings and brought instructions. The informatics nurse would then send email to nurses on the updates’ (N6, female, 28 years).

In the stages leading to the implementation of audit, the team held several discussions about the approaches on which the audit would be framed. Independent and reciprocal roles for each member in the team were clarified. In addition to the directives from the authorities, they conducted literature review to familiarise themselves with standard audit processes to support the decisions. One of such comments was: ‘we recognised the need to search for relevant articles and distribute among ourselves, two members were responsible for reviewing 2 articles. We needed to learn how it was done’ (N14, female, 25 years).

The team reached consensus on the methodology for the audit: ‘we all agreed on sampling methods, inclusion criteria, timeframe for collection and strategies for data analysis’ (N1, female, 34 years). Another participant commented: ‘our aim was to facilitate up-to-date, relevant documentation on Malaffi and paper-based forms and design the methodology of the audit’ (N3, female, 50 years). The hospital documentation guidelines, JCIA standards and SEHA policies were used to develop the standards: ‘attention was given to ensuring that criteria were reasonable and reflected best practice’ (N7, female, 50 years).

The audit instrument developed by the task force focused on the format and structure of documentation, with heavy reliance on the quantity aspects such as the presence of specific data elements. An instruction sheet was developed for scoring: ‘since we were looking at broad issues, we concluded on having one statement per assessment type. The instruction sheet provided a detailed information on scoring’ (N10, female, 41 years). The participants piloted the instrument and the results were analysed using simple descriptive statistics. The instrument was revised two times before it was finalised. The unit health information system link nurse and the chairperson or Designee of the Nursing Documentation Task Force conducted inter-rater reliability. One file was randomly selected from each of the nine units using unique file numbers; the findings were independently subjected to inter-rater reliability testing to establish the level of agreement of interpretation. The time taken to audit one patient record was 45 min. No further reliability analysis was performed. Discrepancies were noted and corrections were made. A new sample was collected after modification of the instrument; 90% level of agreement was achieved.

For the final audit, a proportional number of files were selected using time sampling: ‘we selected the 5th, 10th, 15th and 25th of each month until a total sample size was achieved’ (N12, female, 35 years). Documents of patients with chronic ailments were excluded because of variations in the length of stay (LOS): ‘chronic patients records need to be included, however, we still need to work on long-term care patient documentation guidelines’ (N15, female, 25 years). Both aspects of quantity and quality were addressed in the audit process. However, it was evident that the quantitative aspect that focused on completeness and timeliness was a dominant component. The participants acknowledged that patients’ preferences were not included as the focus of the audit was mostly on completeness and timeliness.

The participants indicated that an instruction tool was developed in accordance with the hospital documentation guidelines to bring clarity on the audit process:

‘The instruction sheet described the scores on the tool, for example, pain assessment must be 100% complete within 24 hours of admission and ongoing assessment must be done whenever change was observed in the status of the patient. Fall risk and nutritional status assessment must be done upon admission and before discharge.’

‘We looked for presence of information about specific care topic to assign a score’ (N2, female, 33 years). The data analysis was also simple descriptive analysis showing frequencies of complete documents.

The length of the audit for one record varied considerably between the initial and final instruments: ‘the final tool took 30 min. We would prefer to do it in less time. However, we are getting good insights from this process’ (N16, female, 50 years). The results of audits showed steady improvements in compliance as stipulated in the hospital guidelines, with the exception of the admission documentation that remained under 90%: ‘we are happy with the results of our audits; however, we would like to achieve the standards as per expectations in the hospital to ensure 100% patient safety’ (N10, female, 41 years). Participants recognised the need for sharing findings of the audit with staff nurses in a transparent and frank manner. ‘We discussed the results with the staff nurses and they suggested some improvements on the audit’ (N8, female, 42 years). They also indicated that nurses whose documentation was being audited expressed concerns regarding the ratings, as the scale indicated whether an intervention was complete or not, but did not show ‘how complete’. By the time this study was conducted, the team was busy reviewing the recommendations from staff nurses.

### Theme 2: Evaluation of audit process

The audit team identified opportunities in using a standardised audit instrument. However, they also noted some challenges. The benefits of a standardised audit tool were listed as the ‘uniformity of audits across units and ability to make comparisons’. The benefits of structured documentation in the EHR were acknowledged, as structured fields allowed for faster review: ‘the templates are good because we could review the record faster during audits, although we need to navigate different windows’ (N4, female, 34 years). However, they acknowledged that electronic documentation is complex. The different locations or fields for data sets make audit tedious. The following statements capture their concerns: ‘the Adhoc and Iview folders for different sets of assessments pose challenges, there are three different application access for nurses such as Power Chart Powerforms and Surginet’ (N16, female, 50 years). ‘When we review patients’ records, there is a nagging question whether or not our findings reflect the true picture of care provided’ (N15, female, 25 years). They also expressed appreciation to the staff nurses who supported the audit process: ‘the staff were able to point out correctly where to locate each component of assessment as we have done with the auditor during Joint Commission International accreditation’ (N11, female, 42 years).

The participants expressed concerns over the amount of documentation required and the duplication of information that ‘they cannot change. However, there are plans now to review the assessment requirements’ (N13, female, 25 years). System-related factors were also cited as a hindrance to audits: ‘downtime periods and absence of save button’ (N15, female, 25 years). Another participant said: ‘interruptions in the system and frequent system updates’ (N2, female, 33 years). Organisational challenges involved using paper-based audits for electronic documentation and various directives from authorities. ‘The constant changes and modifications in documentation requirements meant that we need to constantly revise the criteria for the audit’ (N2, female, 33 years). Nevertheless, the challenges experienced did not seem to reduce the commitment participants demonstrated to learn more and improve their audits.

### Theme 3: Measures to improve documentation audit

As the audit process had a developmental aspect to it, the participants agreed that training needs to be tailored to address specific needs: ‘the ability to provide focused training for the audit team to get a better understanding of protocols and perhaps navigate the electronic record better’ (N5, female, 35 years). Also, they experienced some weaknesses in the dissemination of audit results and suggested focused training: ‘the audit process can be greatly improved once we feel more confident in sharing the results and recommending solutions to documentation’ (N4, female, 34 years).

The participants also addressed the need to have protective time to audit: ‘I usually come during my off duty days to complete the audit, I cannot do the audit when I am doing patient care’ (N9, female, 32 years). Another participant added: *‘*I cannot stay after hours to complete audit as this will incur overtime, we need to have dedicated time for audit’ (N6, female, 28 years).

## Discussion

The findings of this study highlighted the views and experiences of the nurses regarding the implementation of documentation audit in this hospital. Nurses took the lead and participated in quality management processes to improve documentation, and subsequently patient outcomes. However, the task force that was established was not purely for the specific audit project. This was the hospital documentation team that had other roles. Nursing has an obligation to the public to develop measures for the quality of care to enhance patient safety and efficiency of the system (Müller-Staub et al. 2009). They understood the relationship between the documentation, quality, documentation guidelines and audits. The absence of audit models in the region did not deter attempts to improve documentation in this hospital. The fact that another nursing documentation instrument has been developed and implemented implies that the audit of documentation is significant (Björvell et al. 2000). The establishment of a documentation team enabled a much focused and dedicated approach to audit. The participants acknowledged their shortcomings and recognised the importance of conducting literature reviews to obtain in-depth understanding of the audit process. There are different approaches to audit processes; in addition, there is paucity of examples of standardised tools (Johnson et al. 2010; Ritchie et al. 2014; Wang et al. 2011). Clinical audits must be conducted in a professional manner, and the audit team needs specific competencies. Therefore, literature review was the appropriate way to start.

There was a plan agreed upon regarding leadership and roles of the members. The audit aims, objectives and methodology were clearly described and measurable. However, patients’ experiences of the assessment were not included in the audit criteria. The aim of the audit included the improvement of current documentation. The preparation for the audits seemed to have followed all relevant and significant processes involved in developing a reasonable and usable audit for nursing assessment documentation. Their audit cycle was consistent with international standards advocated by Johnson et al. (2013), Wang et al. (2011) and NICE (2002). The audit used two quality criteria: completeness and timeliness for the dimensions of data quality. They are used to measure quality documentation guidelines and SEHA policies for quality and risk management reporting provided the framework to develop standards and criteria. However, this stage could be improved to strengthen criteria. It implies that the hospital guidelines and policies provided evidence that was used as reference, and it was easier for the team to make comparisons. Undertaking a literature review and obtaining expert advice from key stakeholders are two critical steps in the early design process because they provide evidence for practice, thereby contributing to the content validity of a tool (Ritchie et al. 2014). Rating scales used by the team need to be addressed to clarify scoring of elements present, to provide valid results of the quality of documentation. Several researchers posit that review criteria for the clinical audits need to be adequate, appropriate and prioritised according to the quality of evidence from systematic literature reviews (Bravata et al. 2007; Hearnshaw et al. 2002; Johnson et al. 2010; Müller-Staub et al. 2009).

Data sources included patient electronic records; routine data from patient assessments were used. Data collection seemed to have followed their audit plan and parameters for the audit were clearly specified. There was a fair evidence of reliability and validity tests, which indicated room for improvement to ensure the validity of audit results. It was evident that perspective audit required resources in terms of time, as the nurses seemed to have multiple roles. Nevertheless, they were able to provide realistic descriptions of the current documentation practices. Ward or unit nurses participated in the audit although into limited extent; Setz and D’Innocenzzo (2009) argue for greater involvement of nurses in all stages of audits as it facilitates behaviour change. The audit was a priority as per hospital protocols as well as accreditation agencies. The perceived variations in the protocols from authorities meant several reviews and modifications of criteria. The benefits of using a standardised audit tool were acknowledged. Lavin, Harper and Barr (2015) posit that appropriate quality care comparisons among and between providers and practices can only be made when standardised processes and products are used. Standards are important to maximise compatibility and repeatability to achieve high degree of uniformity (Lavin et al. 2015; Mykkänen et al. 2012). The authors believe that mechanisms could be instituted to address variability in the protocols, and this would require increased collaborations with senior management in the hospital.

The team appeared to have completed the last stages of the audit process by comparing the collected data with the standards and guidelines, shared the findings with nursing staff and incorporated their recommendations. They showed the ability to reflect on the strengths and weaknesses of the audit process and suggested measures for improvement. Stage 4: making improvements and stage 5: sustaining improvement appeared to have not been undertaken vigorously. Implementation of audit results and sustaining improvements are areas that require to be strengthened. The integration of results into the hospital quality improvement initiatives could be improved.

## Conclusion

Various strategies are applied to monitor the quality of documentation in nursing practice; documentation audit is one of the important techniques in quality improvement processes. Nurses described their participation in the documentation audit. Data showed that the audit process employed followed the general audit framework; it provided structure and content for data collection, analysis and presentation. The preparation and planning for the audit were found to be areas of strength.

This study revealed several facilitators in the implementation of documentation audit by this team. Irrespective of the level of confidence or competence they verbalised, there was good planning, and the aim and methodology of the audit were well articulated. Strong reliance on literature supported the decisions the team undertook to enhance reliability and validity of the audit process and tool.

The standards and policies from the hospital and SEHA provided the framework for the audit criteria. These were used as benchmarks to assess the quality of documentation. Thus, there was acceptable organisational support for the audit. The participants in this study demonstrated commitment regardless of the multiple roles and time constraints. This study found that nurses were generally content with the guidelines and the audit process they adopted. They reported some successes in that the subsequent audits showed some improvements in the documentation. Conversely, there were challenges such as continuous updates in the documentation procedures, which necessitate modification of the audit tool. The complexity of electronic documentation in relation to paper-based audits is acknowledged. This study showed the significance of documentation audit in ensuring completeness of patients’ records, the role of nurses in audits and the opportunity audits provide to compare documentation guidelines with the quality of documentation in nursing practice.

### Recommendations

To facilitate quality of documentation audits, there must be adequate preparation in terms of training audit personnel and time made available for audits. The organisational support is imperative to successful audits; the design of electronic platforms for documenting patient care can be tailor-made to support electronic data collection for audits. This will shorten the time used to audit the records. The current 30 min per record is not ideal; it takes valuable time off from other important tasks.

This study has demonstrated that nurses recognise the importance of their role in quality management initiatives in the hospital. Therefore, a focused training programme would be beneficial for audit teams to ensure that each stage of the audit is well planned and executed, at the end there would be a structured strategy to implement changes, written reports are disseminated timely to the stakeholders and the plan for the evaluation would be in place. Data from the participants showed there were some weaknesses in these last stages.

### Study limitations

This article reports on one aspect of the audit approach, the team’s experiences and practices of audits. Documentary analysis of the audit instrument is excluded. It would have been beneficial if the study was conducted after the evaluation of documentation audit by the accreditation agency to compare the study findings with the agency’s results.
